# Biological activity of chitosan inducing resistance efficiency of rice (*Oryza sativa* L.) after treatment with fungal based chitosan

**DOI:** 10.1038/s41598-021-99391-w

**Published:** 2021-10-14

**Authors:** Vethamonickam Stanley-Raja, Sengottayan Senthil-Nathan, Kanagaraj Muthu-Pandian Chanthini, Haridoss Sivanesh, Ramakrishnan Ramasubramanian, Sengodan Karthi, Narayanan Shyam-Sundar, Prabhakaran Vasantha-Srinivasan, Kandaswamy Kalaivani

**Affiliations:** 1 Division of Biopesticides and Environmental Toxicology, Sri Paramakalyani Centre for Excellence in Environmental Sciences, Manonmaniam Sundaranar University, Alwarkurichi, Tirunelveli, Tamil Nadu 627 412 India; 2 Department of Biotechnology, St. Peter’s Institute of Higher Education and Research, Avadi, Chennai, Tamil Nadu 600 054 India; 3 Post Graduate and Research Centre, Department of Zoology, Sri Parasakthi College for Women, Courtallam, Tirunelveli, Tamil Nadu 627 802 India

**Keywords:** Plant sciences, Zoology, Chemical biology, Biocatalysis, Chemical ecology, Enzymes, Mechanism of action, Ecology, Behavioural ecology, Biodiversity, Evolution, Origin of life

## Abstract

Reduced pathogen resistance and management of the left-over rice stubble are among the most important challenges faced in rice cultivation. A novel and eco-friendly strategy to synthesise ‘Fungal Chitosan’ (FC) from *Aspergillus niger* using rice straw could serve as a sustainable treatment approach to improve both disease resistance and yields, while also effectively managing the rice stubble waste. The FC treatment promoted germination as well as growth parameters in rice varieties, TN1 (high yielding-susceptible) and PTB33 (low yielding-resistant) better than a commercial chitosan (PC). Treatments of exogenously applied FC to plants produced direct toxicity to *Xoo*, and reduced the BLB disease index by 39.9% in TN1. The capability of FC to trigger a cascade of defense pathways was evident from the measurable changes in the kinetics of defense enzymes, peroxidase (POD) and polyphenol oxidase (PPO). FC treatment increased levels of POD in TN1 by 59.4%, which was 35.3% greater than that of untreated PTB33. Therefore, the study demonstrated the effectiveness of FC treatments for use in agriculture as a potential biostimulant as well as protective agent against bacterial leaf blight, BLB, of rice (*Oryza sativa*) that could be produced from stubble waste and improve rice stubble management strategies.

## Introduction

Rice (*Oryza sativa* L., *Poaceae*) is a vital cereal, grown in a wide array of ecosystems, as one of the top sustaining foods for the global population^1^. As the need for rice increases, so does the stress for farmers and agriculturalists to meet production demands. The major stress comes from trying to protect the crop from pathogen caused diseases and insect pests^2^. However, the greatest dependency is on chemicals to control pests, weeds, and pathogens. These chemical treatments are expensive, and unfortunately an over-dependence upon them often results in the rapid development of resistance among the pests and pathogens^3,4^. Compounding yield losses across all crops are severe changes in climatic conditions, i.e. rainfall, droughts, temperature rise, reduced soil health, or reduced plant vigour, which exacerbates severe yield losses through biotic and abiotic^5^. Therefore, as resistance develops in pests and pathogens, alternatives need to be developed^6^. Crops started from sowing seeds directly into the field are further restricted by poor germination rates^7^ and early infection by pathogens^8,9^. Additionally, seeds with less vigour are under greater stress in adapting to field conditions^10^. But, developing an appropriate pre-treatment, or priming, with a bio-stimulant could laterally enhance the plant’s immune defences, and increase resistance to pests^6^ and pathogens leading to increase yields^11^.

Therefore, the seed germination and triggering of pest/pathogen resistance could be enhanced with several chemical products deployed in agriculture. These include urea and potassium nitrate to amino acids, plant hormones and reactive oxygen–nitrogen–sulphur^12,13^. However, some of these chemical treatments have posed negative effects on the micro and macro environmental fauna^14^, and this increases the impacts from bacterial leaf blight (BLB) one of the most devastating rice pathogens, *Xanthomonas oryzae* pv. *oryzae* (*Xoo*) that causes severe yield losses^4^. Therefore, to counteract the infection, several chemical insecticides have been intensively applied globally^4^, resulting in environmental and health risks along with the development of pest resistance, and reduction of natural enemies^6^. Henceforth exogenous applied alternatives that act as plant stimulants, of biological origin which may be chemicals, proteins, nucleic acids, or microbes (bacteria or fungi) are increasingly important for improving agricultural plant immunity and tolerance to pests and pathogens^4,6,11^.

The term “chitosan” does not describe a unique compound, but a group of commercially available copolymers that produce a heterogeneous variety of molecules depending upon how they are processed during production^15,16^. The wide variety of physical properties across these various forms of chitosan, thus provide molecules for many biological applications in agriculture, bio-fertilizer, foods, pharmacology, medicine, biotechnology and industrial processes^11,16–20^. In rice protection, treatments with chitosan were shown to prevent the growth of several pathogenic bacteria including the rice sheath blight pathogen, *Rhizoctonia solani*^21^, and *Xanthomonas* in the ornamental plant *Euphorbia pulcherrima*, *Xanthomonas axonopodis* pv*. poinsettiicola*^22^.

Available in the markets as a product, commercial chitosan is sourced from crustacean shells^15^ being the second most renewable carbon source after lignocelluloses biomass, with over 1600 tons of chitin annually produced^23^. Although the crustacean based chitosan produces a quality homogenous product with consistent properties and activity it is dependent upon the seafood industry waste stream^15,23^. Thus, alternative production methods using fungal mycelium are focused on providing unlimited sources of chitosan for fermentation and food technologies^16,17,24^.

A result of rice being a highly cultivated crop, is post-harvest rice stubble management, which is turning out to be a menace due to the increase in open-field burning practices that escalates the carbon footprint^25,26^. Therefore, examination of alternative chitosan sources for agricultural uses led to the practice using cellulolytic fungi in the degradation of rice straw, as a post-harvest treatment for stubble management. The implication of using the fungal biomass from the cellulase producing fungi, *Aspergillus niger* could provide a rich source of chitosan production^27^.

This study focussed on synthesising eco-friendly chitosan from *A. niger* using rice straw as a substrate, along with evaluation of its bio stimulant and crop protection competencies in rice plants [BLB susceptible (TN1) and disease resistant (PTB33) varieties].

## Results

### Chitosan characterization

**Figure 1:**
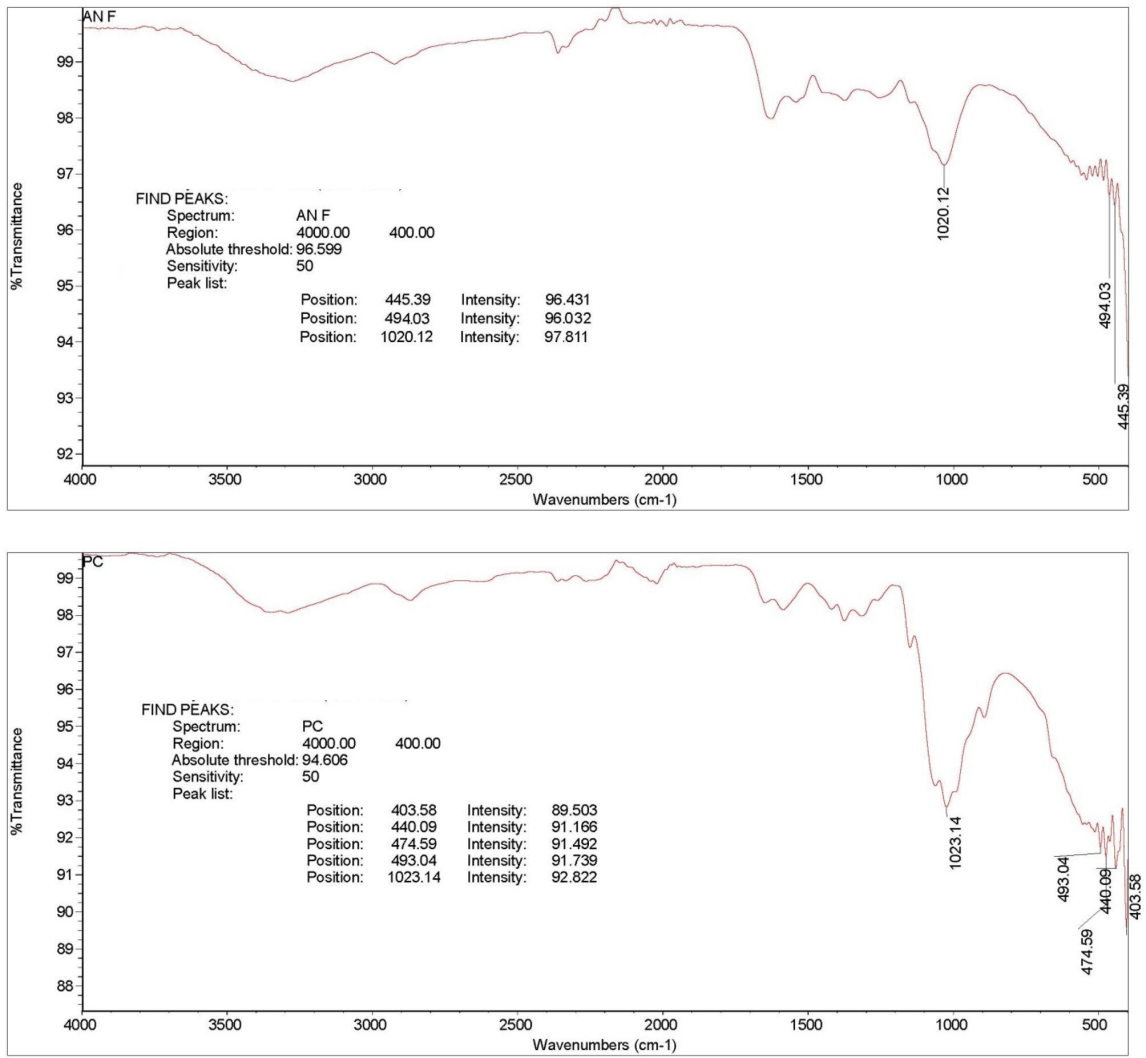
FT-IR spectra. (**A**) Fungal chitosan (FC); (**B**) product chitosan (PC). Similar stretches at wavelength at 3200–3400 cm^−1^ and 2850–3100 cm^−1^ shows the presence of OH and C–H in both PC and FC.

**Figure 2:**
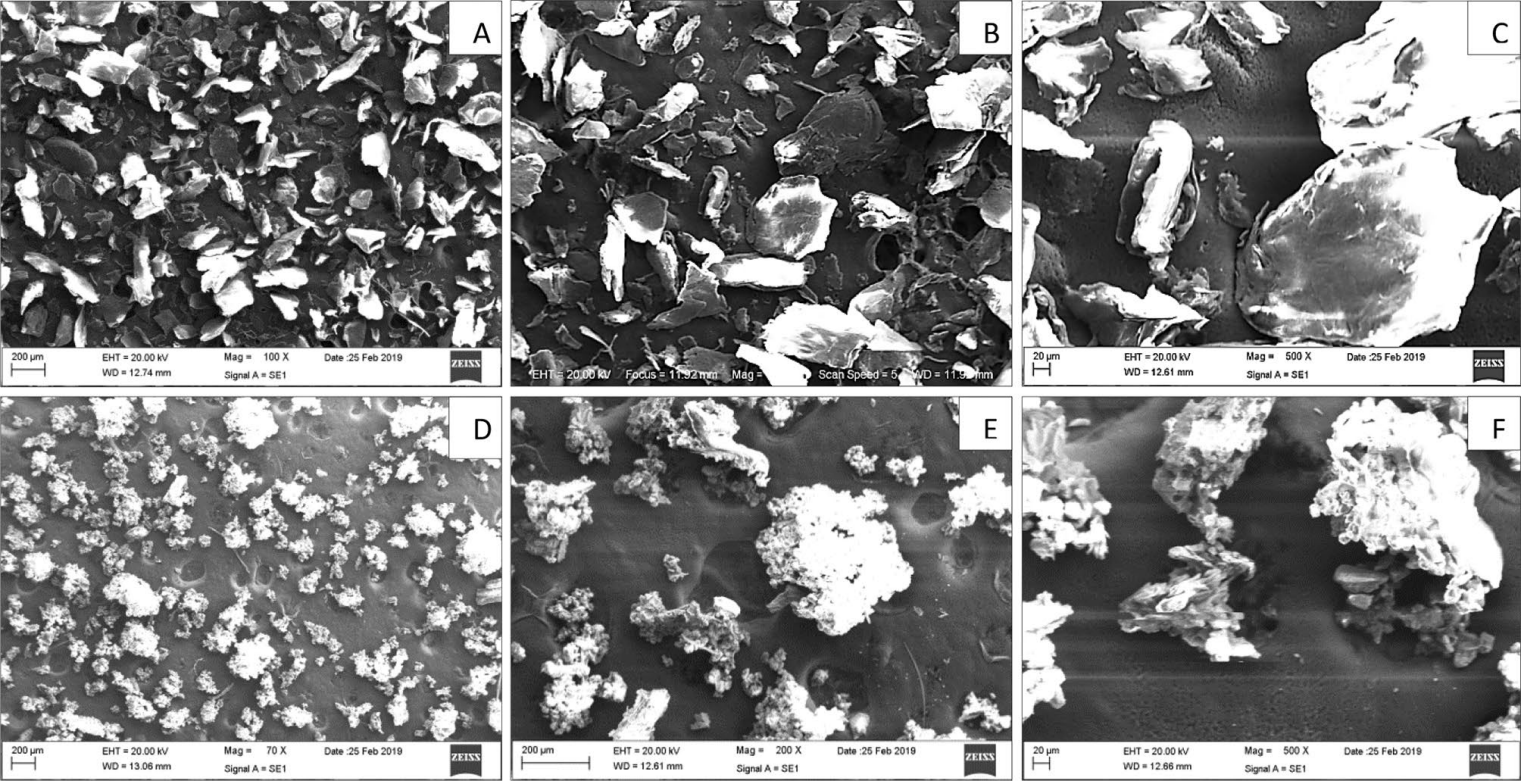
Scanning electron microscope (SEM) images of chitosan; (**A**–**C**) FC 100×, 200×, 500× (**D**–**F**) PC 100×, 200×, 500×.

The extracted fungal chitosan, FC, was analysed in FT-IR and compared with the standard chitosan. The same patterns of peaks were observed in both product chitosan and extracted fungal chitosan. The peaks obtained were between the wavelength at 3200–3400 cm^−1^ presence of H-bonded NH_2_ and OH stretching and 2850–3100 cm^−1^ attributes to CH stretching vibrations which corresponds presence of aliphatic group (Figs. 1 and 2).

Both chitosans (FC and PC) examined under the scanning electron microscope exhibited morphologically similarities (Fig. 2).

### Bio-stimulant effect of chitosan on rice seeds

**Figure 3:**
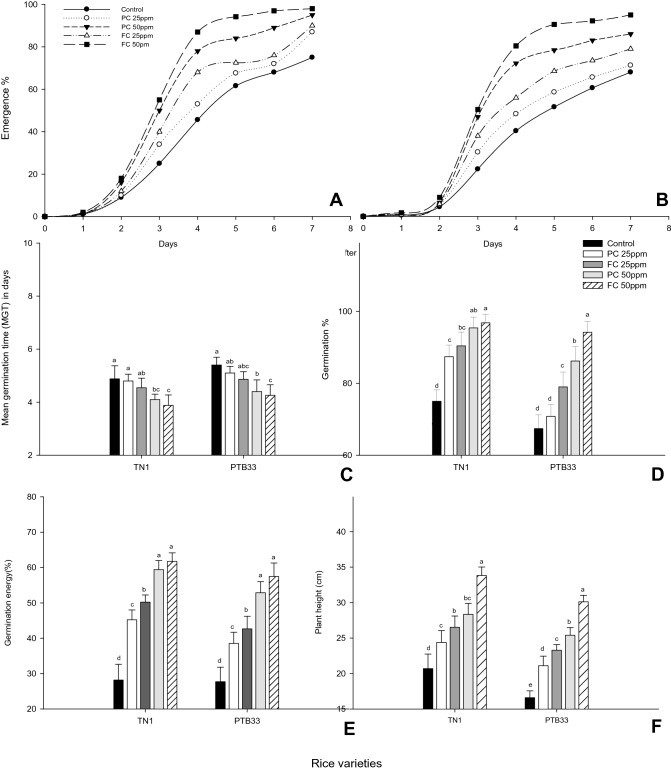
Percentage of emergence of rice varieties treated with fungal chitosan (FC) or the product chitosan (PC) across 12 day period, post treatment (**A**—TN1, **B**—PTB33) (± SEM with five replication) and the. Effect of fungal chitosan (FC) and product chitosan (PC) on mean germination (MGT) days (**C**), percentage of germination (**D**), germination energy (**E**) and plant height (**F**) of rice seeds (± SEM with five replication) (with in the bars denoted by a different letter are significantly different at P ≤ 0.05).

A significant variability in germination parameters was observed in treated seeds from both varieties. Percentage of emergence of rice varieties treated with fungal chitosan (FC) or the product chitosan (PC) across 12 day period, post treatment was found to be higher than that of control rice varieties A-TN1, B-PTB33 (Fig. 3A,B). However, seed treated with fungal chitosan emerged 17 h before that of seeds treated with PC. Both the treatments reduced the mean germination time, promoting the earlier emergence of TN1 that was reduced to 4.54 and 3.8 DAP by PC and FC respectively (*F*_*4,20*_ = 8.87; P ≤ 0.0001). At 50 ppm treatment concentration, PC reduced MGT to 4.86 DAP while FC reduced MGT to 4.26 DAP over untreated control seeds (*F*_*4,20*_ = 8.79; P ≤ 0.0001) (Fig. 3C).

Subsequently there was an increase in germination percentage, GP, over untreated control. The GP of PC and FC treated TN1 seeds at 25 and 50 ppm treatment concentrations were 87.4 and 95.4% and 90.4 and 96.8% respectively (*F*_*4,20*_ = 37.71; P ≤ 0.0001). A likewise increase in germination percentage of PTB33 was also observed at the same treatment concentrations of PC, 86.2% and FC, 94.2% (*F*_*4,20*_ = 44.82; P ≤ 0.0001) (Fig. 3D).

### Bio-stimulant effect of chitosan on germination energy, plant growth and biomass

Increased biomass reported as germination energy, GE, was found to be enhanced by both the treatments over untreated control (26%) (Fig. 3E). PC treatment on TN1 seeds displayed a GE of 59.4% and the GE of FC treated seeds was 61.76 cm at 50 ppm treatment concentration (*F*_*4,20*_ = 102.36; P ≤ 0.0001). A similar effect was also observed in PTB33, resulting in GE of 52.8 and 57.46% respectively treated with 50 ppm PC and FC (*F*_*4,20*_ = 54.86; P ≤ 0.0001).

**Figure 4:**
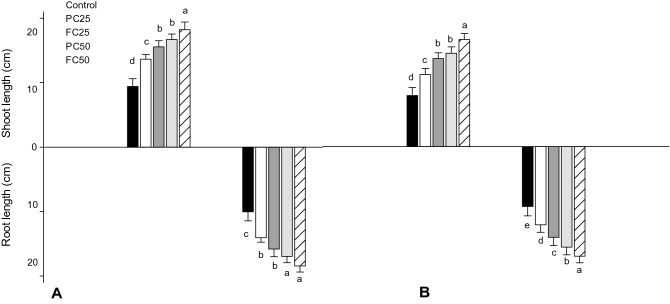
Effect of fungal chitosan (FC) and product chitosan (PC) on mean root and shoot length of rice varieties (**A**—PTB33, **B**—TN1) (± SEM with five replication) (with in the bars denoted by a different letter are significantly different at P ≤ 0.05) [black bars—Control; white bars—PC-25 ppm; dark grey bars—FC-25; light grey bars—PC-50; striped bars—FC-50 ].

**Figure 5:**
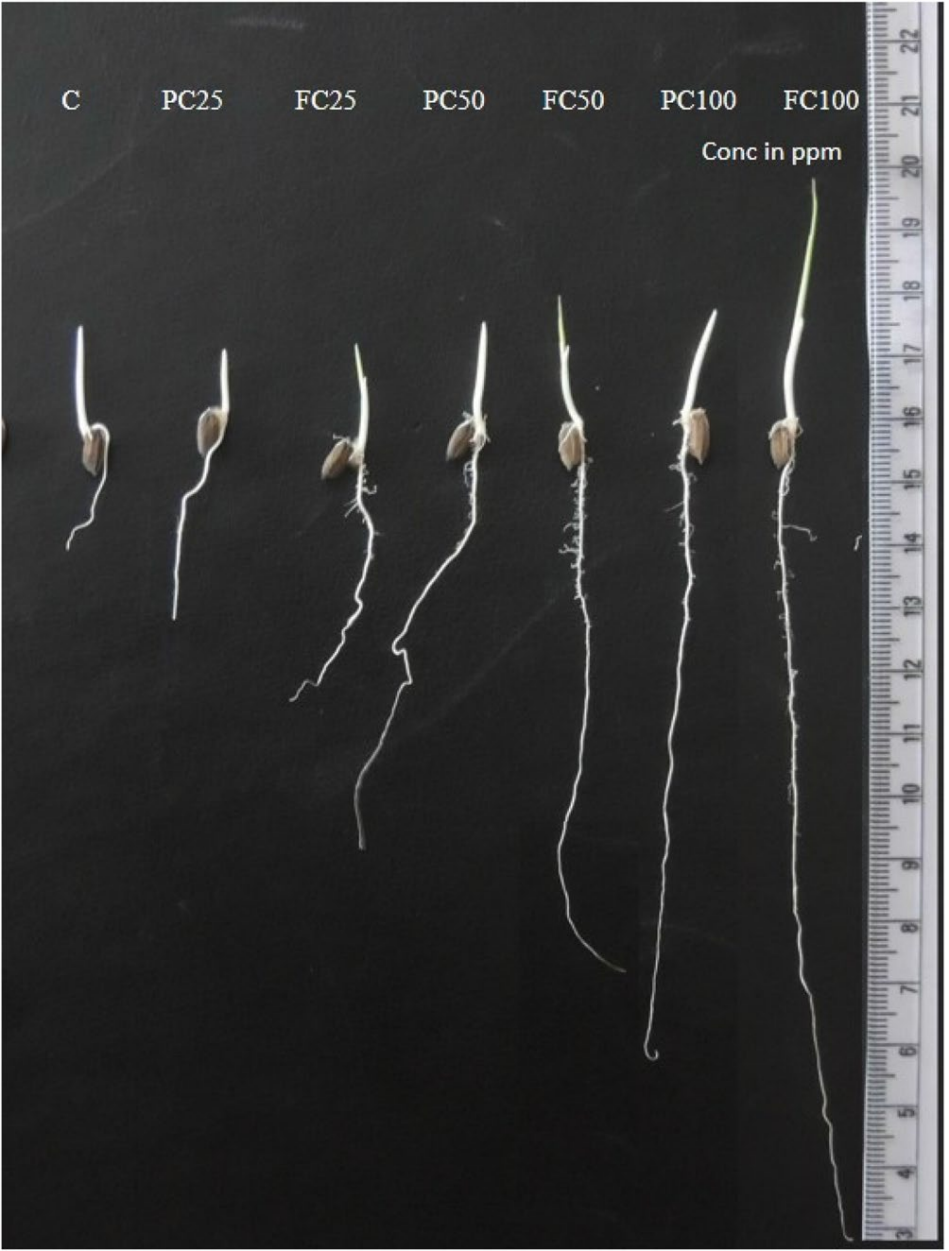
Effect of fungal chitosan (FC) and product chitosan (PC) on Root and Shoot development.

Increased plant growth (height) as an indicator of biomass, shown as germination energy, GE, post treatment with both (PC and FC) was observed at the greatest treatment concentration (50 ppm). Compared to control there were increased plant heights of 28.34 and 33.828 cm with FC (*F*_*4,20*_ = 44.45; P ≤ 0.0001), and 25.4 and 30.126 cm PC (*F*_*4,20*_ = 117.68; P ≤ 0.0001) in TN1 and PTB33 seeds respectively (Fig. 3E). This was due to a 43.5 and 48.31% (*F*_*4,20*_ = 112.38; P ≤ 0.0001) shoot length besides 40.8 and a 45.53% increase in root lengths in TN1 (*F*_*4,20*_ = 68.43; P ≤ 0.0001) (Fig. 3F). Also, greater plant height of PTB33 in PC and FC 50 ppm treatments was attributed to a 45.303 and 52.31% (*F*_*4,20*_ = 101.74; P ≤ 0.0001) increase in shoot lengths along with a 40.38 and 45.35% (*F*_*4,20*_ = 177.52; P ≤ 0.0001) upsurge in root lengths (Figs. 4 and 5).

**Figure 6:**
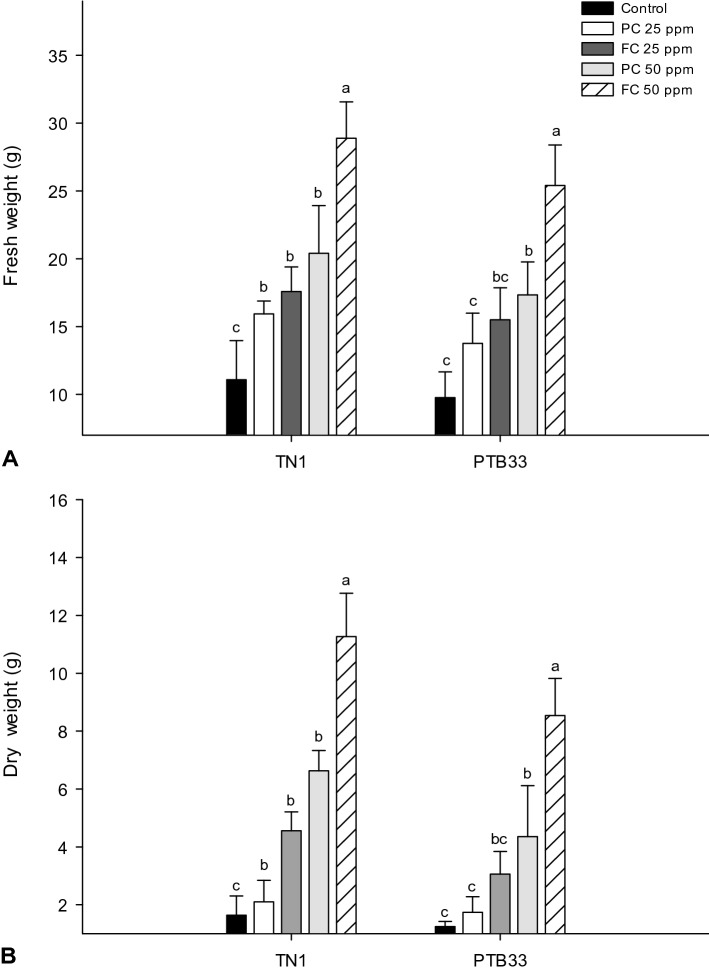
Fresh (**A**) and dry weight (**B**) of rice varieties after treatment with fungal (FC) and product chitosan (PC) (± SEM with five replication) (with in the bars denoted by a different letter are significantly different at P ≤ 0.05).

The FC treatment on PTB33 stimulated growth parameters at a comparatively higher rate than that of TN1 enhancing the biomass of rice plants. The treatments augmented the plant biomass together in both varieties, increasing the FW from 11.08 to 20.41 and 28.88 g (*F*_*4,20*_ = 33.83; P ≤ 0.0001) with a corresponding DW of 6.628 and 11.266 g (*F*_*4,20*_ = 67.9; P ≤ 0.0001) in TN1 treated with 50 ppm of PC and FC respectively (Fig. 6A,B). FC at 50 ppm also induced FW and DW increase from 9.76 to 25.4 g (*F*_*4,20*_ = 28.84; P ≤ 0.0001) and 1.245 to 8.54 g (*F*_*4,20*_ = 37.63; P ≤ 0.0001) respectively which was 31.73 and 48.94% higher than that prompted by PC in PTB33 (P ≤ 0.005) (Fig. 6).

### Antibacterial activity of chitosan against *Xoo*

**Figure 7:**
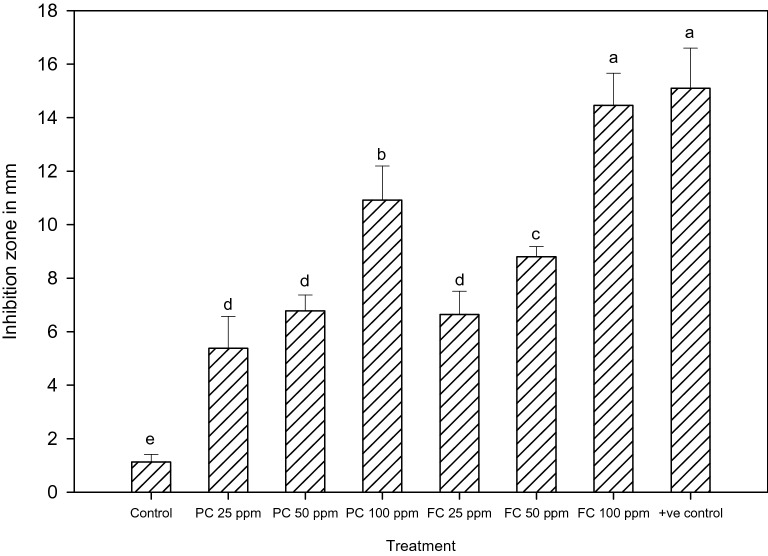
Antibacterial activity of fungal (FC) and product chitosan (PC) (± SEM with five replication) (with in the bars denoted by a different letter are significantly different at P ≤ 0.05).

Both chitosan treatments PC and FC, exhibited significant antibacterial activities that increased with treatment concentrations 25, 50 and 100 ppm (Fig. 7). However the inhibition zones at 25 and 50 ppm were not significant (P ≥ 0.05). At 100 ppm, inhibition zones of both PC (10.2 mm) and FC (14 mm) were significantly different (*F*_*2,12*_ = 70.2; *P* ≤ 0.0001).

### Effect of chitosan spray on BLB disease

**Figure 8:**
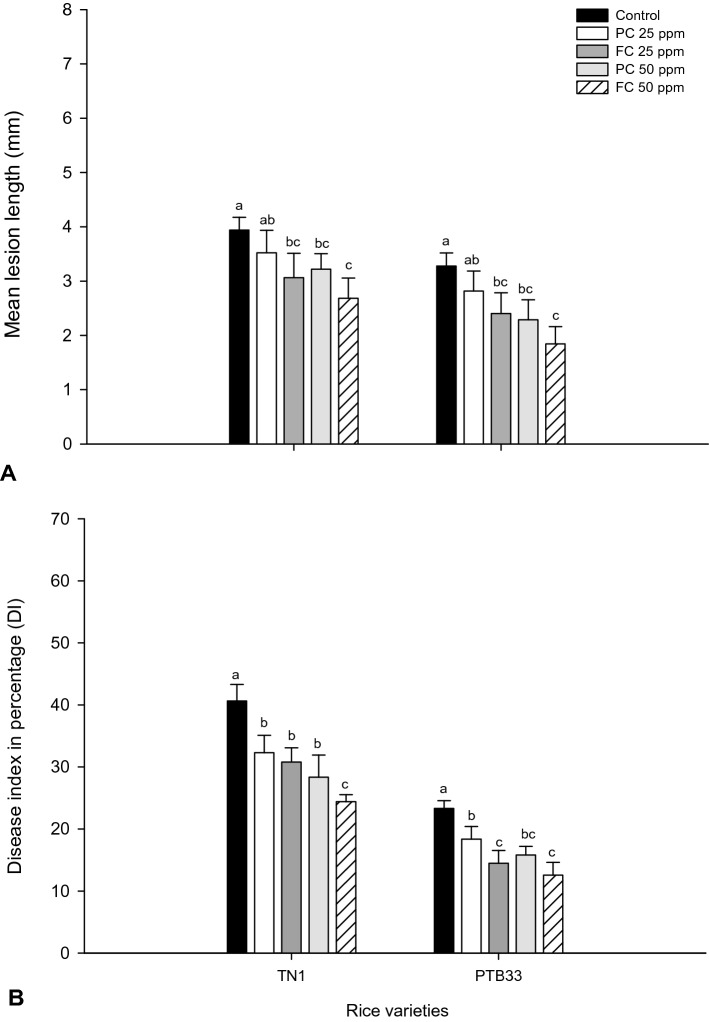
Mean lesion length (cm) (**A**) and disease index (**B**) of *Xoo* after treatment with fungal (FC) and product chitosan (PC) (± SEM with five replication) (with in the bars denoted by a different letter are significantly different at P ≤ 0.05).

The effect of chitosan spray on BLB disease was assessed in terms of mean lesion length and disease incidence percentage. There was a significant reduction in lesion length prompted by chitosan sprays on infected plants (Fig. 8A,B). The lesion length was reduced from 3.9 to 2.8 and 2.4 mm *F*_*4,20*_ = 26.59; P ≤ 0.0001) by PC and FC sprays (50 ppm) in TN1 (Fig. 8A). Control untreated PTB33 plants developed lesions of size 3 mm that were significantly reduce in the the chitosan treatment sprays, PC and FC (50 ppm) to 1.58 and 1.26 mm (*F*_*4,20*_ = 27.25; P ≤ 0.0001). The PC spray (50 ppm) reduced the DI by 30.21 in TN1 and 44.209% in PTB33 (Fig. 8B) compared to control. The 50 ppm FC spray in TN1 reduced the DI to 24.4 from 40.64% (*F*_*4,20*_ = 26.59; P ≤ 0.0001) and to 12.56 from 23.32% (*F*_*4,20*_ = 27.25; P ≤ 0.0001) in PTB33. With originally developed smaller lesions, DI in PTB33 was effectively reduced by 48.52% compared with TN1 by 50 ppm FC spray (P ≤ 0.05) (Fig. 8B).

### Effect of chitosan spray assay on induction of defense enzymes

**Figure 9:**
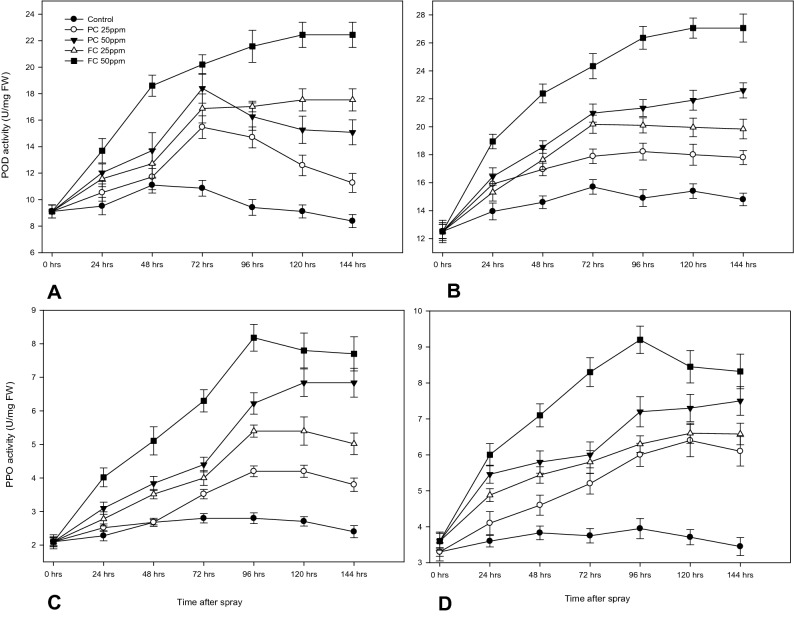
Effects of fungal (FC) and product chitosan (PC) treatments on POD activity in rice plants (**A**—TN1, **B**—PTB33) and on PPO activity in rice plants (**C**—TN1, **D**—PTB33). Data represent the means of five replicates of each treatment.

The effect of chitosan sprays on POD and PPO titers was analysed for across 7 timepoints, starting on day 0 when treated and for 6 days (144 h after spray) (HAS). The enzyme levels differed variably with respect to treatment type, concentration and induction. POD levels treated with 25 ppm PC followed a similar kinetics with that of 50 ppm PC spray in both plant varieties (Fig. 9A,B).

POD levels induced by 50 ppm PC spray on TN1 increased 24 h after spray (HAS) from 9.108 to 12.03 (*F*_*4,20*_ = 25.13; P ≤ 0.0001) and continued to increase to 18.401 U/mg FW till 72 h (*F*_*4,20*_ = 77; P ≤ 0.0001) after which it started to decrease to 15.08 U/mg FW at 144 HAS (*F*_*4,20*_ = 230.74; P ≤ 0.0001) which was still 44.36% higher than control (Fig. 9B). A likely increase in POD levels after 25 and 50 ppm FC sprays displayed an increase in POD levels for 120 HAS after which the levels remained constant till 144 HAS in both plant varieties (Fig. 9A,B). POD levels brought by 50 ppm FC spray on TN1 increased 24 HAS from 9.108 to 13.687 (*F*_*4,20*_ = 25.13; P ≤ 0.0001) and continued to increase to 22.44 U/mg Fresh weight (FW) till 120 HAS (*F*_*4,20*_ = 181.02; P ≤ 0.0001) and staying constant till 144 HAS which was still 32.754% higher than 50 ppm PC spray (Fig. 9A).

Following a similar POD induction kinetics in PTB33, 50 ppm PC spray at 72 HAS raised to 20.18 from 12.513 (*F*_*4,20*_ = 38.65; P ≤ 0.0001) and 22.84 U/mg FW at 144 HAS (*F*_*4,20*_ = 138.01; P ≤ 0.0001). FC spray at 50 ppm, also increased POD levels from 12.513 to a constant level of 27.06 U/mg FW at 144 HAS (*F*_*4,20*_ = 138.01; P ≤ 0.0001) which was 26.68% higher than that induced by 50 ppm PC spray (Fig. 9B). In both the plant varieties, 25 ppm FC spray induced POD levels that were significantly higher than that of 50 ppm PC spray (P ≤ 0.05).

PPO levels of untreated TN1 and PTB33 plants increased till 72 HAS, remained constant till 96 HAS, decreased till 144 HAS (Fig. 9C,D). In TN1, 50 ppm PC spray induced PPO level secretion till 96 HAS to 6.4 U/mg FW (*F*_*4,20*_ = 80.16; P ≤ 0.0001), remaining constant till 120 HAS and declined after that. Yet, TN1 sprayed 50 ppm FC increased PPO secretion till 120 HAS, 7.94 U/mg FW (*F*_*4,20*_ = 75.29; P ≤ 0.0001), remaining constant till 144 HAS (Fig. 9C). FC 50 ppm sprayed PTB33 plants displayed a similar kinetics with the PPO levels raising from 3.6 to 9.22 U/mg FW (*F*_*4,20*_ = 43.5; P ≤ 0.0001) till 120 HAS, remaining constant till 144 HAS (Fig. 9C,D).

## Discussion

Sustainable agricultural practices are of great importance to establish and maintain food security in any country. Use of treatments that improve crop health, reduce pests and pathogens, but that are also environmentally and economically feasible can greatly aid efforts to build sustainable cropping systems. Chitosan demonstrating their capacity to provide an eco-friendly agronomic strategy to improve crop yield and resistance to pathogens and pests^20,28–30^. New methods of chitosan production are providing a renewable and sustainable source of this valuable compounds^15,16,23,24^.

Among them, ligno-cellulose residues are profusely available as an economical viable, natural chitosan resource^31^. Copiously available ligno-cellulose from management of rice stubble using the hydrolytic activity of the fungi, *A. niger* produces an excellent source for the post treatment production of chitosan^32^. This study further shows the benefits from *A. niger* produced chitosan as a seed priming agent to induce improvements in germination capabilities of low yielding, disease resistant, rice variety PTB33. Chitosan produced from *A. niger* treated rice stubble, also demonstrates their capacity to induce BLB disease resistance in high yielding, susceptible TN1 rice variety.

Utilizing the natural cellulosic substrate of rice straw as the source to increase the biomass of *A. niger* and their subsequent chitosan concentration, fungal chitosan was successfully extracted and shown to be comparable in efficacy with commercially available sea-shell chitosan product. Extracting the chitosan from the fungal mycelia using SSF provided the maximal production of hydrolytic enzymes such that rice straw utilization to produce chitosan is economically feasible^33^. The chitosan quality was further supported by analyses using FT-IR spectrum which indicates the strong similarity in the produced and purchased chitosan compositions. Similar analyses using spectral uniformity between commercial and fungal chitosan extracted from *Auricularia *sp., previously reported similar results^34^.

Seed treatment with the chitosan, FC, produced using *A. niger*, conclusively caused improvements in seed germination GP, and energy GE, traits for both varieties, TN1 and PTB33, when using concentration of FC (50 ppm). When using FC priming of PTB33 seeds early germination was observed, reducing the MGT from 5.4 to 4.26 days. Lizárraga-Paulín et al.^35^ reported the major effects of chitosan-seed interactions to be displayed in terms of enhanced germination index, EGI, reduced mean germination time, MGT, and flowering time/number, with augmented growth in height and root length which correlated with increase biomass^35^. Hadwiger et al. showed the transfer of chitosan from seed to seedling also affects the development of seedlings and the post development processes^36^. Germination energy was designated as a parameter of seed quality in different sunflower genotypes^37^. An increase in germination energy indicates the seed vitality, also plays a direct role and are the key factors in determining plant number per hectare and yield.

A lateral increase in the GP and GE positively influenced the establishment capability by producing plants with 44.89% greater lengths, with a corresponding 45% increase in root-shoot lengths, and a 61.57% increase in plant biomass (FW) compared to untreated disease resistant seeds. The FC treatment prompted the growth of PTB33 to be 31.28% greater than untreated TN1. The plant biomass increases and early establishment provoked by activation of various biochemical processes has found to enrich the grain nutrient status^38^. A likely increase in plant vegetative growth by chitosan seed priming was reported by Hameed et al. in wheat seeds^39^. Additionally, the optimistic influence of chitosan on seeds was reported by Zhou et al. in coriander and tomato and by Samarah et al. on pepper^40,41^. The accomplishment of chitosan as a successful seed dress is attributed to their higher molecular weight conferring physical protection^42^. Furthermore, the capability of chitosan to induce the activities of lipase, gibberellic acid and indole acetic acid are endorsed for their active priming properties^40^.

The ability of chitosan to inhibit *Xoo* under in vitro conditions produced a positive outcome. The higher treatment concentrations of FC and PC produced significant differences in bactericidal activities, with FC performing better than PC. Kulikov et al. reported that differences among bioactivities of chitosan rely upon their source, degree of polymerization or type^43^. However, the antibacterial activity of PC and FC did not differ significantly at lower concentrations (25 and 50 ppm). Chitosan was previously documented with antibacterial activities against *Escherichia coli*, *Staphylococcus aureus* along with *Bacillus* sp^44^.

The effect of exogenous application of chitosan to confer resistance against BLB was analysed. The disease index of the chitosan sprayed plants was considerably reduced. The disease control mechanism is an indication of the activation of innate plant defense systems as confirmed by the secretion of pathogenesis related enzymes (POD and PPO). The POD level in TN1 increased by 59.4% when treated with FC, which was 35.3% higher than that of resistant PTB33 at the end of 144 HAS. Analogous increase in PPO levels was also observed in TN1 plants treated with FC. At 50 ppm FC concentration, the PPO level increased 67.3% compared to control and was only 16.78% less than levels in the resistant PTB33 at 144 HAS. The treatments also altered the enzyme kinetics, displaying a continuous rise till 120 HAS and remaining stable after that in both plant varieties. Meanwhile the enzyme levels in leaves treated with PC started to decrease after 120 HAS.

A corresponding chitosan induced disease resistance was reported in treated wheat seeds against Fusarium blight reducing the severity of the disease^45^. Rabea et al. have extensively reviewed the potential of chitosan foliar treatment on the control of various plant pathogens^46^. Despite having the same spectral composition, the ability of FC to outperform PC in terms of conferring disease resistance might be attributed to their ability to induce local and systemic reactions with a build-up of multiple defense-related^47^. The effect of chitosan to modulate plant defense systems in response to various pathogens has been reported and characterised by the accumulation of phytoalexins, pathogenesis related proteins, along with proteinase inhibitors^44^. Apart from being directly toxic to pathogens, chitosan was also found to enhance host resistance in date palm against the wilt pathogen by increasing the synthesis of POD and PPO^47^.

Based upon the results, the ability of chitosan treatments were shown to improve the germination capabilities along with disease resistance of rice plants. In addition to managing the peril of rice stubble, the fungal based chitosan production system clearly improved resistance as a bio-stimulant, and elicitor of the plant defense pathway, producing a better response than the commercial crustacean-based chitosan treatment. Therefore, the application of fungal chitosan in agricultural systems could diminish the undesirable influence of disease-causing pathogens on the produce and quality of rice and other crops, along with providing economic relief to growers using rice stubble for fungal chitosan production as a sustainable agricultural system that is more effective and profitable.

## Materials and methods

### Microorganisms and inoculum preparation

**Figure 10:**
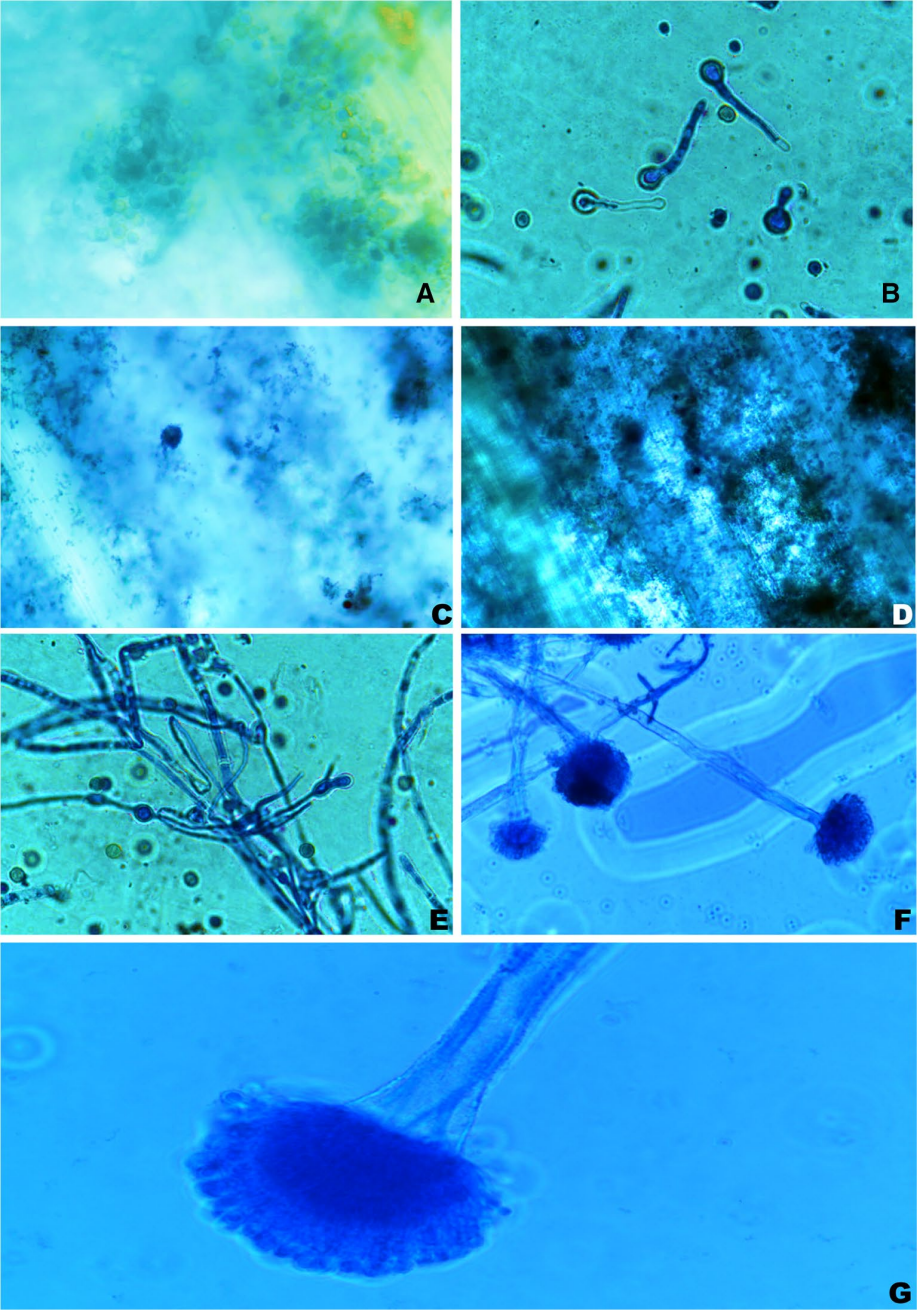
Development of *A. niger* in rice straw after inoculation (**A**—Spores accumulated after inoculation, **B**—*A. niger* spore germination, **C**—Development of mycelia in rice straw, **D**—Thick growth has been seen in the 3rd day after inoculation, **E**—Mycelial growth of *A. niger* on 5th day after inoculation, **F**— 100× magnification of conidiophore along with fungal mycelium, **G**—100× magnification of fruiting body of *A. niger*).

*A. niger* (RHS/M492-NAIMCC-F-02890) was purchased from the National Agriculturally Important Microbial Culture Collection (NAIMCC), Uttar Pradesh, India. The fungus was re-cultured in PDA, maintained in the laboratory (4 °C). Production inoculum was prepared by inoculating fungal spores in Potato Dextrose Broth (PDB-30 ml, pH 5) and incubated (28 °C; 72 h). The spores were harvested and adjusted to 2 × 10^7^ Spores ml^−1^ using 0.1% tween 80 in sterile distilled water by haemocytometer counting^48^. (Figure 10).

*Xoo* culture from previous experiments was used in this study^49^. The bacteria were cultured (Nutrient broth; 48 h), centrifuged (8000×*g*—15 min) and bacterial count was adjusted to 3 × 10^5^ CFU/ml (sterile distilled water; haemocytometer counting).

### Straw pre-treatment and solid-state fermentation (SSF)

The process of SSF was carried out for chitosan extraction from pre-treated straw by modifying the methods of Rane and Hoover and Crestini et al.^50,51^. Rice stubble was collected from local field (Alwarkurichi, Tamil Nadu, India). Appropriate permission was obtained from the agriculture land owners before collecting the stubble. The rice straw was rinsed with tap water, shaken of excess water and then pulverized (size 1–2 cm). A synthetic medium (0.2% yeast extract, 1.0% peptone and 2.0% glucose) was developed to humidify the straw 60% water content^52^. The substrates were autoclaved at 121 °C for 20 min, inoculated with spore suspension in sterile plastic bags and closed with cotton plugs to avoid contamination by preventing air flow, then maintained at 30 °C for 15 days.

### Chitosan extraction

The solid-state biomass were ground and suspended with 1 M NaOH solution and autoclaved at 121 °C for 30 min 1 M NaOH solution and autoclaved at 121 °C for 30 min. Insoluble alkali fractions were collected by centrifugation at 11,600×*g* for 15 min and washed with distilled water, then again centrifuged at neutral pH 7.0. The alkali insoluble residues were centrifuged, freeze dried and weighed. The residues were extracted using 2% acetic acid at 95 °C for 12 h. The slurry was centrifuged at 11,600×*g* for 15 min and the acid insoluble fraction was discarded. The supernatant was collected, adjusting to a pH of 10 using 2 N NaOH producing precipitated alkali insoluble chitosan. The precipitated chitosan was collected and air dried at 60 °C to a calculated weight and deacetylated as in Zhang et al.^53^.

### Preparation of chitosan

Fungal chitosan extracted from *A. niger* and a standard-control Chitosan product (Catalogue no: 448877-50G, Sigma-Aldrich) were used to prepare stock solutions of chitosan (3 mg/mL)—fungal chitosan (FC) and product chitosan (PC—Sigma-Aldrich) (1% acetic acid; pH 6). After stirring (160 rpm) for 24 h at room temperature, the stock solutions were autoclaved at 121 °C for 20 min. Filtered sterilized deionized water (pH 6) was used as a blank-control. The chitosan test concentrations (25 ppm and 50 ppm) were prepared from the FC and PC stock solutions.

### FTIR characterization of chitosan

FT-IR (Fourier Transform Infrared) spectra were recorded for FC on a Perkin Elmer Spectrum One, equipped with an ATR-FTIR (attenuated total reflection-FTIR) unit (16 co-addition scans in a wavelength range of 400–4000 cm^−1^) and compared with readings of the PC.

### Seed collection and preparation

Rice varieties, TN1 (susceptible) and *Xoo* resistant (PTB33) were procured from National Rice Research Institute, Indian Council of Agricultural Research Cuttack (seeds were used for only research purpose with proper permission). Seeds received were of similar dimensions and were surface sterilized prior to use for experiments^54^. Both cultivars mentioned above were used for research purpose only and it does not come under endangered species of wild flora and fauna as per IUCN. Essential methods and guidelines are followed from the IUCN.

### Bio-stimulant effect of chitosan

For the germination assays, 100 seeds of each variety per treatment—FC, PC and control were soaked in 25 ml of FC, PC and sterile distilled water (24 h). Filter paper method was used to analyse the germination parameters inclusive of emergence, germination percentage (GP), mean germination time (MGT) and germination energy (GE)^54^. The experiments were replicated five times to obtain the raw data before processing the statistical analysis.

In germination assay the emerging hypocotyls were recorded every day and the mean germination time (MGT) was premeditated^55,56^ by calculating the time taken for 1, 10, 25, 50, 75 and 100% of the seeds to germinate (expressed as days).

$${\text{MGT}} = \frac{\sum (n T)}{{\sum n}}$$

where n = number of germinated seeds at time T (25 °C). T = hours from the beginning of the germination test. Σn = final germination.

The germination percentage (GP) was calculated using the following formula

$${\text{Germination}}\;{\text{percentage}}\;({\text{GP}}) = \frac{{{\text{Number}}\;{\text{of }}\;{\text{seeds}}\;{\text{germinated}}}}{{{\text{Total}}\;{\text{number }}\;{\text{of}}\;{\text{seeds}}}} \times 100$$

Seed Germination Energy (GE) was calculated according to the formula

$${\text{Seed}}\;{\text{Germination}}\;{\text{Energy}}\;({\text{GE}}) = \frac{{{\text{Number}}\;{\text{of }}\;{\text{germinating}}\;{\text{seeds}}}}{{{\text{No}}{.}\;{\text{of}}\;{\text{total }}\;{\text{seeds }}\;{\text{per}}\;{\text{ test}}\;{\text{ post }}\;{\text{germination }}\,{\text{for }}\;{3}\;{\text{ days}}}} \times 100$$

### Effect of seed treatment under green-house

Rice seeds, TN1 and PTB33 were sown (5 seeds/treatment; 0.5 L pots). The potting soil and experimental conditions were followed by the method of Kalaivani et al.^49^ After 20 days of sowing (DOS), growth parameters (Total plant height, root and shoot length in cm) and biomass (fresh and dry weight—oven drying—40 °C for 2 days) of plants was determined.

### Antibacterial activity of chitosan against *Xoo*

Bacterial suspension (10 µl; 3 × 10^5^ CFU/ml of *Xoo*) was used in disc diffusion method to determine the antibacterial activity of FC, PC (25, 50 and 100 ppm) and sterile distilled water (control)^57^. Inhibition zones were measured in diameter (mm) post incubation (28 ± 2 °C; 48 h). Tetracycline (1 mg/ml) is used as positive control and control (0.1% acetic acid) were used in the assay. Three replication of samples were loaded in respective prelabelled wells to record the zone of incubation. The plates were kept upright position and incubated at 37 °C for 24 h.

### Induced resistance by spray treatment of chitosan against BLB

#### Xoo inoculation

*Xoo* was inoculated on rice plants grown under greenhouse conditions mentioned for seed treatment assay (27–33 °C; 12 h L: D, 90% RH), 28 DAS by scissor-dip method^58^. Symptoms of BLB were observed 7 days post inoculation (DPI).

#### Exogenous application of chitosan

FC and PC (25 and 50 ppm), 15 ml, were sprayed uniformly on the inoculated rice plants rice plants at the maximum seedling stage in green house condition on 15th day after planting. Plants sprayed with sterile distilled water served as untreated control.

#### Disease assessment

Lesion length was measured on 15 DPI and data for one treatment was obtained from 40 inoculated leaves. Subdual of BLB was evaluated in terms of reduction in the mean BLB lesion length^59^.

### Effect of spray treatments on the induction of defense related enzymes

Leaves were analysed at specific time intervals (0, 24, 48, 72, 96, 120 and 144 h) post treatments for the levels of peroxidase (PO) in addition to polyphenol oxidase (PPO) determined by the methods proposed by Hammerschmidt et al.^60^ and Worthington^61^ with five replicates.

### Statistical analysis

Bio-stimulant, antibacterial activity and disease assessment experiments were done with five replicates before undergone the arcsine squire root transformation and other statistical performance. One-way ANOVA (analysis of variance) was performed on the experimental data and treatment means were paralleled by Tukey’s-family error test (P < 0.05) by means of Minitab17 software package. The data were arcsine transformed before undertaking statistical analysis.

## Data Availability

The datasets generated during and/or analyzed during the current study are not publicly available due to funding agency agreement and intellectual properties but are available from the corresponding author on reasonable request with permission of funding agency.

## References

[1] Chaney RL, Kim WI, Kunhikrishnan A, Yang JE, Ok YS (2016). Integrated management strategies for arsenic and cadmium in rice paddy environments. Geoderma.

[2] Nakashima K, Yamaguchi-Shinozaki K, Shinozaki K (2014). The transcriptional regulatory network in the drought response and its crosstalk in abiotic stress responses including drought, cold, and heat. Front. Plant Sci..

[3] Senthil-Nathan S (2013). Physiological and biochemical effect of neem and other Meliaceae plants secondary metabolites against Lepidopteran insects. Front. Physiol..

[4] Kalaivani K, Maruthi-Kalaiselvi M, Senthil-Nathan S (2021). Seed treatment and foliar application of methyl salicylate (MeSA) as a defense mechanism in rice plants against the pathogenic bacterium, *Xanthomonas**oryzae* pv. *oryzae*. Pest Biochem. Physiol..

[5] Das G, Rao GJN (2015). Molecular marker assisted gene stacking for biotic and abiotic stress resistance genes in an elite rice cultivar. Front. Plant Sci..

[6] Senthil-Nathan S (2015). A review of biopesticides and their mode of action against insect pests. Environ. Sustain..

[7] Shi W (2016). Grain yield and quality responses of tropical hybrid rice to high night-time temperature. Food Crop Res..

[8] Farooq M (2011). Rice direct seeding: Experiences, challenges and opportunities. Soil Till. Res..

[9] Brown JKM (2002). Yield penalties of disease resistance in crops. Curr. Opin. Plant Biol..

[10] Liu H (2012). Antifungal effect and mechanism of chitosan against the rice sheath blight pathogen, *Rhizoctonia solani*. Biotechnol. Lett..

[11] Orzali, L., Corsi, B., Forni, C. & Riccinoi, L. Chitosan in agriculture: A new challenge for managing plant disease, biological activities and application of marine polysaccharides. *Biol. Act. Appl. Mar. Polysaccharides*. 17–36. 10.5772/66840 (2017).

[12] Anosheh HP, Sadeghi H, Emam Y (2011). Chemical priming with urea and KNO3 enhances maize hybrids (*Zea**mays* L.) seed viability under abiotic stress. J. Crop Sci. Biotechnol..

[13] Hänsch R, Mendel RR (2009). Physiological functions of mineral micronutrients (Cu, Zn, Mn, Fe, Ni, Mo, B, Cl). Curr. Opin. Plant Biol..

[14] Savvides A, Ali S, Tester M, Fotopoulos V (2016). Chemical priming of plants against multiple abiotic stresses: Mission possible?. Trends Plant Sci..

[15] Kurita K (2006). Chitin and chitosan: Functional biopolymers from marine crustaceans. Mar. Biotechnol..

[16] Hamed I, Özogul F, Regenstein JM (2016). Industrial applications of crustacean by-products (chitin, chitosan, and chitooligosaccharides): A review. Trends Food Sci. Technol..

[17] Badawy MEI, Rabea EIA (2011). Biopolymer chitosan and its derivatives as promising antimicrobial agents against plant pathogens and their applications in crop protection. Int. J. Carbohydr. Chem..

[18] Davydova VN (2011). Chitosan antiviral activity: Dependence on structure and depolymerization method. Appl. Biochem. Microbiol..

[19] Park BK, Kim MM (2010). Applications of chitin and its derivatives in biological medicine. Int. J. Mol. Sci..

[20] Malerba M, Cerana R (2016). Chitosan effects on plant systems. Int. J. Mol. Sci..

[21] Liu H (2014). Progress and constraints of dry direct-seeded rice in China. J. Food Agric. Environ..

[22] Li B, Wang X, Chen R, Huangfu W, Xie G (2008). Antibacterial activity of chitosan solution against *Xanthomonas* pathogenic bacteria isolated from *Euphorbia pulcherrima*. Carbohydr. Polym..

[23] Falcón-Rodríguez, A. B., Cabrera, J. C., Wégria, G., Onderwater, R. C. A., González, G., Nápoles, M. C., Costales, D., Rogers, H. J., Diosdado, E., González, S., Cabrera, G., González, L. & Wattiez, R. Practical use of oligosaccharins in agriculture. In Ist World Congress on the use of biostimulants in agriculture. *Acta Hortic*. **1009**, 195–212 (2012).

[24] Yin H (2016). Genome shuffling of *Saccharomyces cerevisiae* for enhanced glutathione yield and relative gene expression analysis using fluorescent quantitation reverse transcription polymerase chain reaction. J. Microbiol. Methods.

[25] Borah N (2016). Low energy rice stubble management through in situ decomposition. Procedia Environ. Sci..

[26] Singh R, Srivastava M, Shukla A (2016). Environmental sustainability of bioethanol production from rice straw in India: A review. Renew. Sustain. Energy Rev..

[27] Mrudula S, Murugammal R (2011). Production of cellulase by *Aspergillus niger* under submerged and solid state fermentation using coir waste as a substrate. Braz. J. Microbiol..

[28] El-Sayed SM, Mahdy ME (2015). Effect of chitosan on root-knot nematode, *Meloidogyne javanica* on tomato plants. Int. J. ChemTech Res..

[29] Iriti M, Varoni EM (2015). Chitosan-induced antiviral activity and innate immunity in plants. Environ. Sci. Pollut. Res..

[30] Orzali L (2017). Chitosan in agriculture: A new challenge for chitosan in agriculture: A new challenge for managing plant disease managing plant disease. InTech Open Publisher.

[31] Nanda S, Mohammad J, Reddy SN, Kozinski JA, Dalai AK (2014). Pathways of lignocellulosic biomass conversion to renewable fuels. Biomass Convers. Biorefinery.

[32] Aggarwal NK, Goyal V, Saini A, Yadav A, Gupta R (2017). Enzymatic saccharification of pretreated rice straw by cellulases from *Aspergillus niger* BK01. 3 Biotech.

[33] Fatma, H., Abd-EI-Zaher & Fadel, M. Production of bioethanol via enzymatic saccharification of rice straw by cellulase produced by *Trichoderma Reesei* under solid state fermentation. *N. Y. Sci. J.*, 72–78. http://www.sciencepub.net/newyork (2010).

[34] Chang AKT, Frias RR, Alvarez LV, Bigol UG, Guzman JPMD (2019). Comparative antibacterial activity of commercial chitosan and chitosan extracted from *Auricularia* sp. Biocatal. Agric. Biotechnol..

[35] Lizárraga-Paulín EG, Miranda-Castro SP, Moreno-Martínez E, Lara-Sagahón AV, Torres-Pacheco I (2013). Maize seed coatings and seedling sprayings with chitosan and hydrogen peroxide: Their influence on some phenological and biochemical behaviors. J. Zhejiang Univ. Sci. B..

[36] Hadwiger LA, Fristensky B, Riggleman RC (1984). Chitosan, a natural regulator in plant-fungal pathogen interactions, increases crop yields. Chitin Chitosan Relat. Enzymes..

[37] Mrda J, Crnobarac J, Dušanić N, Jocić S, Miklič V (2011). Germination energy as a parameter of seed quality in different sunflower genotypes. Genetika.

[38] Singh H (2015). Seed priming techniques in field crops—A review. Agric. Rev..

[39] Hameed A, Sheikh MA, Farooq T, Basra SMA, Jamil A (2013). Chitosan priming enhances the seed germination, antioxidants, hydrolytic enzymes, soluble proteins and sugars in wheat seeds. Agrochimica.

[40] Zhou YG, Yang YD, Qi YG, Zhang ZM, Wang XJ, Hu XJ (2002). Effects of chitosan on some physiological activity in germinating seed of peanut. J. Peanut Sci..

[41] Samarah NH, Wang H, Welbaum GE (2016). Pepper (*Capsicum annuum*) seed germination and vigour following nanochitin, chitosan or hydropriming treatments. Seed Sci. Technol..

[42] Chen JL, Zhao Y (2012). Effect of molecular weight, acid, and plasticizer on the physicochemical and antibacterial properties of β-chitosan based films. J. Food Sci..

[43] Kulikov SN, Chirkov SN, Il’ina AV, Lopatin SA, Varlamov VP (2006). Effect of the molecular weight of chitosan on its antiviral activity in plants. Appl. Biochem. Microbiol..

[44] El Hadrami A, Adam LR, El Hadrami I, Daayf F (2010). Chitosan in plant protection. Mar. Drugs.

[45] Orzali L, Forni C, Riccioni L (2014). Effect of chitosan seed treatment as elicitor of resistance to *Fusarium graminearum* in wheat. Seed Sci. Technol..

[46] Rabea EI, Badawy MET, Stevens CV, Smagghe G, Steurbaut W (2003). Chitosan as antimicrobial agent: Applications and mode of action. Biomacromol.

[47] Wang X, El Hadrami A, Adam LR, Daayf F (2008). Differential activation and suppression of potato defence responses by *Phytophthora infestans* isolates representing US-1 and US-8 genotypes. Plant Pathol..

[48] Smits JP, Rinzema A, Tramper J, Schlösser EE, Knol W (1996). Accurate determination of process variables in a solid-state fermentation system. Process Biochem..

[49] Kalaivani K, Kalaiselvi MM, Senthil-Nathan S (2016). Effect of methyl salicylate (MeSA), an elicitor on growth, physiology and pathology of resistant and susceptible rice varieties. Sci. Rep..

[50] Rane KD, Hoover DG (1993). An evaluation of alkali and acid treatments for chitosan extraction from fungi. Process Biochem..

[51] Crestini, C., Kovac, B. & Giovannozzi-Sermanni, G. Production of chitosan by fungi. **50**, 207–210. 10.1002/bit.260500202 (1996). 10.1002/bit.260500202 18626937

[52] Khalaf SA (2004). Production and characterization of fungal chitosan under solid-state fermentation conditions. Int. J. Agric. Biol..

[53] Zhang ZT, Chen DH, Chen L (2002). Preparation of two different serials of chitosan. J. Dong Hua Univ. Engl. Ed..

[54] Chanthini KM, Stanley-Raja V, Thanigaivel A, Karthi S, Palanikani R, ShyamSundar N, Sivanesh H, Soranam R, Senthil-Nathan S (2019). Sustainable agronomic strategies for enhancing the yield and nutritional quality of wild tomato *Solanum Lycopersicum* (l) Var Cerasiforme Mill. Agronomy.

[55] Ellis RH, Roberts EH (1980). Improved equations for the prediction of seed longevity. Ann. Bot..

[56] Chanthini KM (2019). Biocatalysis and agricultural biotechnology *Chaetomorpha**antennina* (Bory) Kützing derived seaweed liquid fertilizers as prospective bio-stimulant for *Lycopersicon**esculentum* (Mill). Biocatal. Agric. Biotechnol..

[57] Murray PR, Baron EJ, Pfaller MA, Tenover FC, Yolke RH (1995). Manual of clinical Microbiology.

[58] French ER (1986). Efficacy of five methods of inoculating potato plants with *Pseudomonas solanacearum*. Phytopathology.

[59] Yasmin S (2017). Biocontrol of Bacterial Leaf Blight of rice and profiling of secondary metabolites produced by rhizospheric *Pseudomonas**aeruginosa* BRp3. Front. Microbiol..

[60] Hammerschmidt R, Kuć J (1982). Lignification as a mechanism for induced systemic resistance in cucumber. Physiol. Plant Pathol..

[61] Worthington CC (1988). Worthington Enzyme Manual: Enzymes and Related Biochemicals.

